# Upregulation of miR-196b Confers a Poor Prognosis in Glioblastoma Patients via Inducing a Proliferative Phenotype

**DOI:** 10.1371/journal.pone.0038096

**Published:** 2012-06-19

**Authors:** Ruimin Ma, Wei Yan, Guojun Zhang, Hong Lv, Zhizhong Liu, Fang Fang, Wei Zhang, Junxia Zhang, Tao Tao, Yongping You, Tao Jiang, Xixiong Kang

**Affiliations:** 1 Laboratory Diagnosis Center, Beijing Tiantan Hospital, Capital Medical University, Beijing, China; 2 Department of Neurosurgery, Beijing Tiantan Hospital, Capital Medical University, Beijing, China; 3 Department of Neurosurgery, The First Affiliated Hospital of Nanjing Medical University, Nanjing, China; University of Florida, United States of America

## Abstract

**Purpose:**

To explore the expression pattern, prognostic value and functional role of miR-196b in glioblastoma (GBM) patients using large cohorts.

**Experimental Design:**

MiR-196b expression was measured using the Human v2.0 miRNA Expression BeadChip (Illumina) in 198 frozen glioma tissues. The expression levels of miR-196b were also validated in an independent cohort containing 128 formalin-fixed paraffin-embedded (FFPE) glioma samples using qRT-PCR. The presence of other molecular prognostic indicators was assessed centrally in the glioma samples. Whole genome gene profiling was performed to investigate the underlying biological behavior. MiR-196b functional analyses were performed in U87 and U251 cell lines.

**Results:**

The expression levels of miR-196b were inversely correlated with overall survival in GBM patients. Gene set enrichment analysis (GSEA) showed that the gene sets relating to cell cycle were significantly enriched in the cases with miR-196b overexpression. Functional analyses in U87 and U251 cells revealed that miR-196b was involved in cell proliferation.

**Conclusions:**

MiR-196b is overexpressed and confers a poor prognosis via promoting cellular proliferation in GBM patients.

## Introduction

Glioblastoma (GBM) is the most aggressive type of glioma. Though there have been advances in therapy, the prognosis for a GBM patient is still dismal, and the patients have a median survival of only 14.6 months [1], [2]. Furthermore, GBM patients show remarkably different survival times from less than one week to more than three years following diagnosis [3]. However, to date, only the IDH1 mutation and G-CIMP signature have been validated to predict prognosis across various studies [4], [5].

MicroRNAs (miRNAs) belong to a recently discovered class of small non-coding RNA molecules that regulate the expression of multiple target genes and multiple cellular processes, including cell differentiation, stem cell maintenance, and epithelial–mesenchymal transition [6]. Beyond their involvement in a variety of biological processes, miRNAs have also been found to participate in processes involved in the molecular pathology of cancer [7]. Depending on the genes targeted, miRNAs can act either as oncogenes or tumor suppressors [8]. It should be noted that miRNAs are very stable molecules that can exist in FFPE samples without degradation for a long period of time and can potentially be specific prognostic markers to be used in clinic [9]. Recently, some studies have identified that specific miRNA expression signatures can provide insight into the diagnosis and prognosis of a variety of human cancers [10]–[12]. It has been reported that there is a relationship between miRNA expression and the outcome of GBM patients [13]. However, to date, no stable prognostic marker based on miRNA expression has been reported in GBMs across various institutes.

Previous studies have shown contradictory conclusions regarding the expression patterns and prognostic values of miR-196b in GBM patients [14], [15]. Here, we report the expression pattern of miR-196b using the expression values from microarrays of 198 frozen glioma tissues. Furthermore, the expression pattern and prognostic value of miR-196b were validated in an independent cohort containing 128 FFPE samples. In addition, to underscore the potential biological insights of miR-196b, 60 GBM samples were subjected to whole genome gene profiling. Integrated analysis of miR-196b and whole genome gene expression patterns showed that overexpression of miR-196b was tightly correlated with the gene sets related to cell cycle. Functional assays showed that miR-196b might serve as a potential target for anti-proliferation therapies in GBM patients.

## Materials and Methods

### Samples and Patients

All glioma samples included in our study were from the Chinese glioma genome atlas (CGGA). The patients underwent surgical resection between January 2006 and December 2009 and subsequently received radiation therapy or alkylating agent-based chemotherapy. Patients were eligible for the study if the diagnosis of glioma was established histologically according to the 2007 WHO classification. Ten normal brain tissues were included. Of them, seven normal adult brain samples were obtained after informed consent from patients with severe traumatic brain injury who needed post-trauma surgery and three other normal samples were from patients who had undergone surgery for primary epilepsy. This study was approved by the institutional review boards of Beijing Tiantan Hospital and The First Affiliated Hospital of Nanjing Medical University, and written informed consent was obtained from all patients.

**Figure 1:**
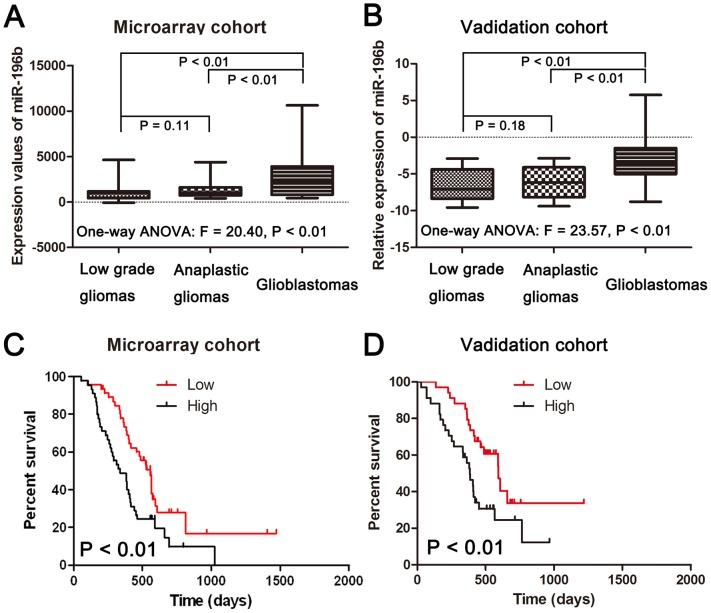
The expression of miR-196b in gliomas and its association with survival in GBMs. (A) Expression patterns of miR-196b in the microarray cohort containing 198 frozen glioma tissues. (B) Expression patterns of miR-196b in the validation cohort containing 128 glioma FFPE glioma tissues. (C) Kaplan-Meier survival curves according to the expression of miR-196b in 91 frozen GBM tissues. (D) Kaplan-Meier survival curves according to the expression of miR-196b in 68 FFPE GBM tissues. The log-rank test was used to calculate p values.

### RNA Extraction

All of the tissue samples were immediately snap-frozen in liquid nitrogen after surgery. A hematoxylin and eosin stained frozen section was prepared for assessment of the percentage of tumor cells before RNA extraction. Only samples with greater than 80% tumor cells were selected. Total RNA from frozen tumor tissues was extracted using the mirVana miRNA Isolation kit (Ambion, Austin, TX, USA) according to the manufacturer’s protocol. RNA concentration and quality were measured using the NanoDrop ND-1000 spectrophotometer (NanoDrop Technologies, Houston, TX, USA). MiRNAs of FFPE tissues were extracted using an RNeasy FFPE Kit (QIAGEN).

### MiR-196b Expression Analyses

MiR-196b expression values of 198 glioma tissues were from Chinese Glioma Genome Atlas (CGGA), which is a database that focuses on glioma. Briefly, 200 ng of total RNA was polyadenylated and then reverse transcribed into to cDNA using a biotin-labeled Oligo dT primer with a universal PCR sequence. After cDNA synthesis, miRNAs were individually interrogated using specific oligonucleotides. A single miRNA-specific Oligo (MSO) was designed against each mature miRNA sequence, and miRNA-specific primers were extended using DNA polymerase. Universal primers were used to amplify the cDNA templates, and the primer complimentary to the array was fluorescently labeled. Finally, the labeled, single-stranded PCR products were hybridized to the Human v2.0 miRNA Expression BeadChip (Illumina, Inc., San Diego, CA, USA) with 1,146 human miRNAs (97% coverage of the miRBase 12.0 database).

**Figure 2:**
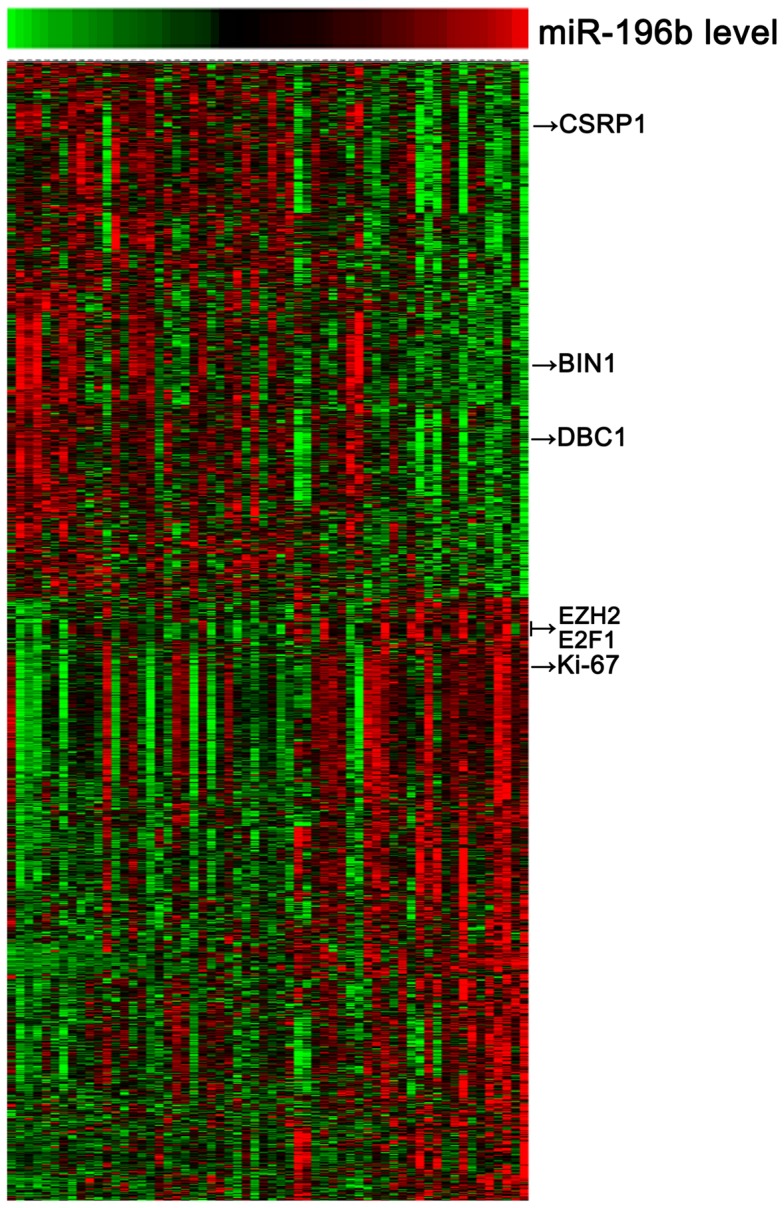
Heat map of the gene-expression signature correlated with miR-196b expression. Rows represented probe sets and columns represented patients. Patients were ordered from left to right by increasing miR-196b expression. Expression levels of the probe sets were represented by color, with green demonstrating expression less than and red demonstrating expression greater than the median value for the given probe set. Arrows indicated genes that were discussed in the text.

**Table 1:** Gene sets enriched in GBM samples with miR-196b overexpression.

NAME	ES	NES	NOM p-val	FDR q-val
CELL_CYCLE_PROCESS	−0.47827	−2.57053	0	0
CELL_CYCLE_PHASE	−0.4891	−2.55122	0	0
M_PHASE	−0.5207	−2.54711	0	0
MITOSIS	−0.52103	−2.46316	0	0
M_PHASE_OF_MITOTIC_CELL_CYCLE	−0.52028	−2.4344	0	0
MITOTIC_CELL_CYCLE	−0.46339	−2.41896	0	0
CELL_CYCLE_GO_0007049	−0.40213	−2.31263	0	8.40E-05
MITOTIC_SISTER_CHROMATID_SEGREGATION	−0.70647	−2.21738	0	4.91E-04
CHROMOSOME_SEGREGATION	−0.56145	−2.15355	0	9.87E-04
CHROMOSOME_ORGANIZATION_AND_BIOGENESIS	−0.43082	−2.1452	0	9.98E-04

**Figure 3:**
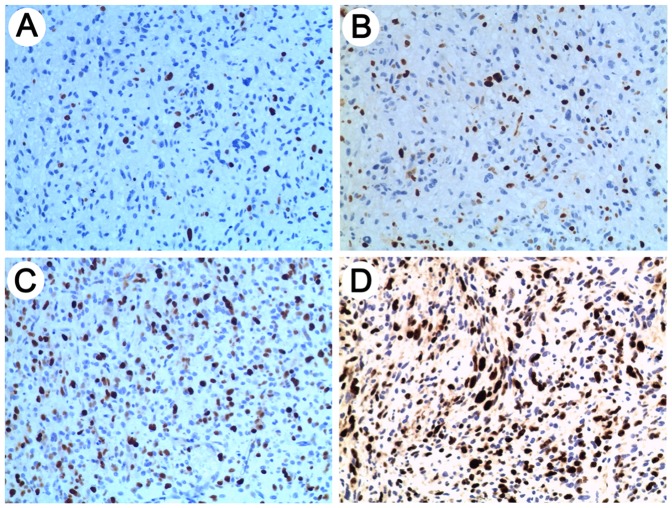
Representative antibody stainings for Ki-67. IHC results of Ki-67 are shown on a scale of 0 to 3 (0, <10% (A); 1, 10%–30% (B); 2, 30%–60% (C); 3, >60% (D)). And scale of 0 and 1 and scale of 2 and 3 indicated low and high expression, respectively.

### Whole Genome Gene Profiling

Microarray analysis was performed using the Agilent Whole Human Genome Array according to the manufacturer’s instructions. The integrity of the total RNA was checked using an Agilent 2100 Bioanalyzer (Agilent, Santa Clara, USA). cDNA and biotinylated cRNA were synthesized and hybridized to the array. Data were acquired using the Agilent G2565BA Microarray Scanner System and Agilent Feature Extraction Software (v9.1). Probe intensities were normalized using GeneSpring GX 11.0.

**Table 2:** Correlation of miR-196b and Ki-67 expression.

	High miR-196b	Low miR-196b
High expression of Ki-67 protein	32 (47%)	17 (25%)
Low expression of Ki-67 protein	2 (3%)	17 (25%)

Pearson correlation: R = 0.492, P<0.01.

**Figure 4:**
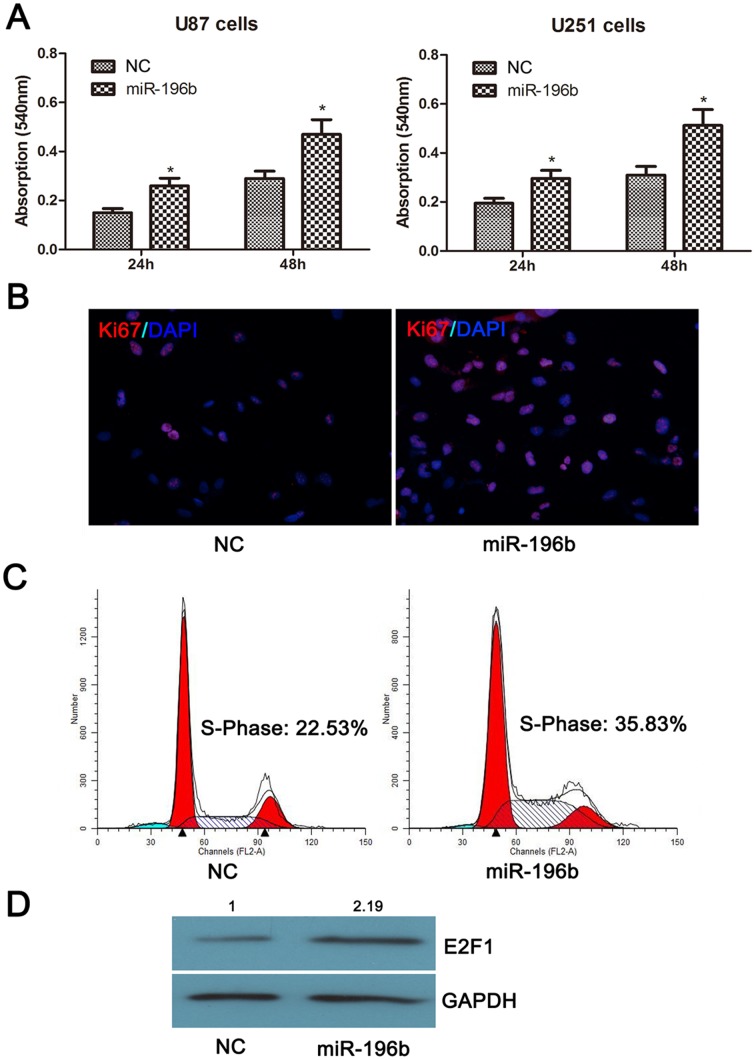
MiR-196b functions as an oncogene via promoting cell proliferations in glioma cells. (A) miR-196b could increased proliferation in U87 and U251 cells. (B) miR-196b could increase the fraction of Ki-67+U251 cells. (C) miR-196b could increase S-Phase proportion in U251 cells. (D) miR-196b could increase E2F1 expression at protein level in U251 cells. *p<0.05.

### Immunohistochemistry

Immunohistochemistry was performed as described in the previous report [16]. Briefly, surgical specimens were fixed in formalin, routinely processed and paraffin embedded. Five-micron-thick sections were prepared, and immunohistochemical staining with streptavidin-biotin immunoperoxidase assay was performed using antibodies against Ki-67. The staining intensity was scored by two pathologists without knowledge of clinical information on a scale of 0 to 3 (0, negative; 1, slight positive; 2, moderate positive; 3, intense positive). A score of 0 and 1 or 2 and 3 indicated low or high expression, respectively. Controls without primary antibody and positive control tissues were included in all experiments to ensure the quality of staining.

### Gene set Enrichment Analysis

To obtain more information about the biologic processes related to miR-196b expression in GBM, we performed gene-expression profiling using Agilent Whole Human Genome Array and GSEA as described previously [17].

### Cell Culture and Oligonucleotide Transfection

U87 and U251 glioma cells were purchased from the Chinese Academy of Sciences Cell Bank. SHG44 cells were cultured in DMEM culture medium supplemented with 10% fetal bovine serum (FBS). All cells were maintained in a 37°C, 5% CO2 incubator and routinely passaged at two-to three-day intervals. Cells from passages 2–4 were used in all experiments.

Oligonucleotide transfection was performed using mimics of miR-196b and mimics negative control (NC) that were chemically synthesized by Shanghai GenePharma Company (Shanghai, China). Cells were transfected using LipofectAMINE 2000 reagent (Invitrogen, Carlsbad, USA). Transfection complexes were prepared according to the manufacturer's instructions and added directly to the glioma cells to result in a final oligonucleotide concentration of 10 nmol/L. The transfection medium was replaced 8 h post-transfection.

### Cell Growth Assays

The MTT assay was used to determine relative cell growth. U87 and U251 cells were plated at 10^4^ cells per well in 96-well plates with six replicate wells for each condition, transfected and assayed 24 h and 48 h post-transfection. A cell growth assay was performed using MTT (Sigma, St. Louis, MO) as described in ref [18]. The cell viability was determined at 540 nm absorbance using an enzyme-linked immunosorbent assay plate reader. All data points represent the mean of a minimum of six wells.

### Western Blotting Assays

As described in our previous report [19], to determine the levels of E2F1 expression, total protein was isolated in lysis buffer (137 mM NaCl, 15 mM EGTA, 15 mM MgCl2, 0.1 mM sodium orthovanadate, 0.1% Triton X−100, 25 mM MOPS, 100 µM phenylmethylsulfonyl fluoride, and 20 µM leupeptin, adjusted to pH 7.2). Equal amounts of protein (30 µg) were loaded into the sample wells and separated on a 12% sodium dodecyl sulfate-polyacrylamide gel. The electrophoresed proteins were transferred to a polyvinylidene difluoride Immobilon-P membrane (Millipore, Watford, UK) and subjected to immunoblot analysis with the respective antibodies. GAPDH was reblotted to check for equal loading of the gel.

### Cell Cycle Assay

U251 cells transfected with miR-196b and NC for 48 h were collected, washed in phosphate-buffered saline (PBS), and fixed with 70% cold ethanol for at least 1 h. After extensive washing, the cells were suspended in HBSS containing propidium iodide (69 µM; Sigma) and RNase A (0.1 mg/mL; Sigma), incubated for 1 h at room temperature, and analyzed by FACScan (Becton Dickinson) [20].

### Immunofluorescence

U251 cells transfected with miR-196b and NC for 48 h were seeded on coverslips and fixed with 4% paraformaldehyde, treated with 3% H2O2 for 10 min, blocked for nonspecific staining with 10% bovine serum albumin (Sigma-Aldrich) in PBS for 1 h and incubated with the Ki-67 antibody (1∶40 dilutions) overnight at 4 °C. TRITC-conjugated Goat Anti-Mouse antibody (1∶200 dilutions) was added for 2 h at 37 °C. DAPI reagent was used to stain the U251 cell nuclei and the cells were visualized using an immunofluorescence microscope and analyzed using IPP5.1 (Olympus).

### Statistical Analysis

Kaplan-Meier survival analysis was used to estimate the survival distributions, and the log-rank test was used to assess the statistical significance between stratified survival groups using GraphPad Prism 5.0 statistical software. Student's t-tests, One-way ANOVA and Exact Sig (2-sided) χ2 test were performed using SPSS 13.0. All data are presented as the means ± SE. A two-sided P value of <0.05 was considered significant.

## Results

### MiR-196b is Upregulated and Confers a Poor Prognosis of GBM Patients

The expression of mature miR-196b was measured in a series of 198 glioma samples (63 low-grade gliomas, 44 anaplastic gliomas and 91 GBMs) via microarrays. As shown in Figure 1A, GBMs demonstrated a significant increase in miR-196b transcript levels compared to the mean expression levels observed in low-grade gliomas (p<0.01) or anaplastic gliomas (p<0.01). No significant difference in miR-196b expression levels was observed between low-grade and anaplastic gliomas (P = 0.11). To further confirm this result, we performed qRT-PCR to examine miR-196b levels in an independent cohort containing 128 FFPE samples (30 low grade gliomas, 30 anaplastic gliomas and 68 GBM samples). As shown in Figure 1B, miR-196b was significantly upregulated in GBMs compared to that of low-grade gliomas (p<0.01) or anaplastic gliomas (p<0.01). No difference in miR-196b expression levels was observed between low-grade and anaplastic gliomas (P = 0.18). Furthermore, we found that the differential expression of miR-196b between normal tissues and low grade and anaplastic gliomas is not statistical significant (P = 0.84 for low grade gliomas; P = 0.19 for anaplastic gliomas). However, normal tissues have a significant lower of miR-196b when compared glioblastoma tissues (P = 0.01) (Figure S1). The correlation between miR-196b expression and overall survival was measured through Kaplan-Meier survival curve analysis with a log-rank comparison. GBM samples expressing higher than median levels of miR-196b were associated with decreased survival relative to those with miR-196b levels lower than the median (p<0.01) in the microarray cohort (Figure 1C). Further, miR-196b expression was inversely correlated with overall survival in the 68 GBM samples of the independent validation set (p<0.01) (Figure 1D). These results also demonstrated that miR-196b was stable in FFPE slides and might have potential for future clinic use.

### MiR-196b is Tightly Associated with the Biological Process of the Cell Cycle

To gain insights into the biologic processes related to miR-196b expression in GBMs, sixty GBM samples with large variability in miR-196b transcript levels were subjected to whole genome gene profiling. A total of 1294 probes significantly correlated (R>0.3 or <−0.3, P<0.01) with miR-196b expression levels (Figure 2; Table S1; 685 probes with positive correlation and 609 with negative correlation).Arrows indicate the genes that are discussed in the text. Gene set enrichment analysis (GSEA) showed that the gene sets related to cell cycle were significantly enriched in the cases with miR-196b overexpression (Table 1). Using a p value of <0.001, 23 biologic processes mainly related to cell cycle, M phase, mitosis, and chromosome segregation were significantly enriched in the GBM samples with miR-196b overexpression. Furthermore, the immunohistological staining of proliferation marker Ki-67 set was performed in the 68 GBM samples of the independent validation. A positive correlation of miR-196b and Ki-67 was also found (Figure 3; Table 2).

### MiR-196b Functions as an Oncogene by Enhancing Cell Proliferation in GBMs

To analyze the functional role of miR-196b in GBMs, we overexpressed miR-196b using transient transfection in U87 and U251 cells. Changes in cell proliferation were assessed at 24 and 48 h post-transfection. The U87 and U251 cells transfected with miR-196b mimics showed increased cell proliferation rates compared to those of mock-transfected cells (Figure 4A). Further functional experiments were performed in U251 cells. As shown in Figure 4B and Figure 4C, miR-196b could increase the fraction of Ki−67+U251 cells and S-phase proportion of U251 cells. Besides, miR-196b could increase E2F1 expression at protein level in U251 cells (Figure 4D). These results indicated that miR-196b might be a potential target for anti-proliferation therapy in GBMs.

## Discussion

It has been reported that aberrant miRNA expression patterns highly correlate with progression and prognosis in various cancers. MiRNAs are very stable in FFPE tissues on slides, with no degradation over a long period of time, and are thus a potential diagnostic and prognostic tool in clinic [21]. In the present study, we investigated the expression level of miR-196b in two independent cohorts, totaling over 300 glioma patients. We further showed that the expression level of miR-196b was upregulated and conferred a poor prognosis in GBM patients. Whole genome gene profiling was performed to investigate the underlying biological behavior. MiR-196b functional analyses were performed in U87 and U251 cell lines.

The expression pattern and functional role of miR-196b are very controversial across various cancers. Several studies have demonstrated that miR-196b functions as a tumor suppressor in leukemia [22]–[26]. The expression of miR-196b has also been reported to be significantly elevated in gastric cancer [27]. To date, there are two articles that have reported the correlation of miR-196b with prognosis in GBMs, but they draw opposite conclusions. Guan *et* *al.* reported that miR-196 is overexpressed and negatively correlated with OS [15]. However, the opposite association between miR-196b and OS was reported in GBM patients in another institute [14]. The numbers of GBM patients in the above two studies was 39 and 38, respectively. The large variability of results may be due to the small sample numbers. Larger studies need to be performed to establish the expression pattern and prognostic significance of miR-196b in GBM patients. Here, we evaluated the expression of miR-196b in 198 frozen glioma samples using microarray. Furthermore, we validated the expression pattern of miR-196b in 128 glioma FFPE tissues. We found that miR-196b was significantly overexpressed in GBMs when compared with low-grade and anaplastic gliomas. However, there is no significant difference in miR-196b levels between low grade and anaplastic gliomas. Meanwhile, survival analysis revealed that overexpression of miR-196b in 91 frozen GBM samples conferred a poor prognosis. The prognostic value of miR-196b was also validated in 68 FFPE GBM samples. These results indicated that miR-196b might be involved in malignant progression and function as potential oncogene in GBMs.

To date, no study has been performed to investigate the biological processes of miR-196b in GBMs systematically. In the present study, sixty GBM samples with large variability in miR-196b transcript levels were subjected to whole genome gene profiling. We observed a negative correlation of miR-196b expression with a series of tumor suppressor genes, includingCSRP1, DBC1 and BIN1 [28]–[30]. Some oncogenes that drive cell proliferation, including E2F1 and EZH2, were also found to positively correlate with the expression of miR-196b [31], [32]. The proliferation marker Ki-67 also showed a positive correlation with miR-196b expression. GSEA analysis showed that the gene sets related to cell cycle were significantly enriched in the cases with miR-196b overexpression. Further in vitro experiments showed that the transfection of miR-196b mimics into glioma cells could increase cell proliferation significantly. These results suggest that miR-196b functions as an oncogene via promoting cell proliferation in GBMs.

Our results show that miR-196b is overexpressed in GBMs and confers poor prognosis based on large samples in both frozen and FFPE samples. We also show that miR-196b is tightly associated with cell cycle progression and that ectopic expression in GBM cells could increase proliferation significantly. In summary, unlike in other cancers, miR-196b is an oncogene and could be a potential target for anti-proliferative therapy in GBMs.

## Supporting Information

Figure S1 **MiR-196b expression levels in 10 Normal tissues, 15 low grade gliomas, 15 anaplastic gliomas and 15 glioblastomas were evaluated by real-time qRT-PCR.** And we found that the differential expression of miR-196b between normal tissues and low grade and anaplastic gliomas is not statistical significant (P = 0.84 for low grade gliomas; P = 0.19 for anaplastic gliomas). However, normal tissues have a significant lower of miR-196b when compared Glioblastoma tissues (P = 0.01).
(TIF)

Table S1 **A list of the 1294 probes that correlate with expression of mir-196b.**
(XLSX)
