# Supplementary figures and images for: Upregulation of miR-196b Confers a Poor Prognosis in Glioblastoma Patients via Inducing a Proliferative Phenotype
Source: PLoS One. 2012 Jun 19;7(6):e38096. doi: 10.1371/journal.pone.0038096 (PMC3378534; doi:10.1371/journal.pone.0038096)

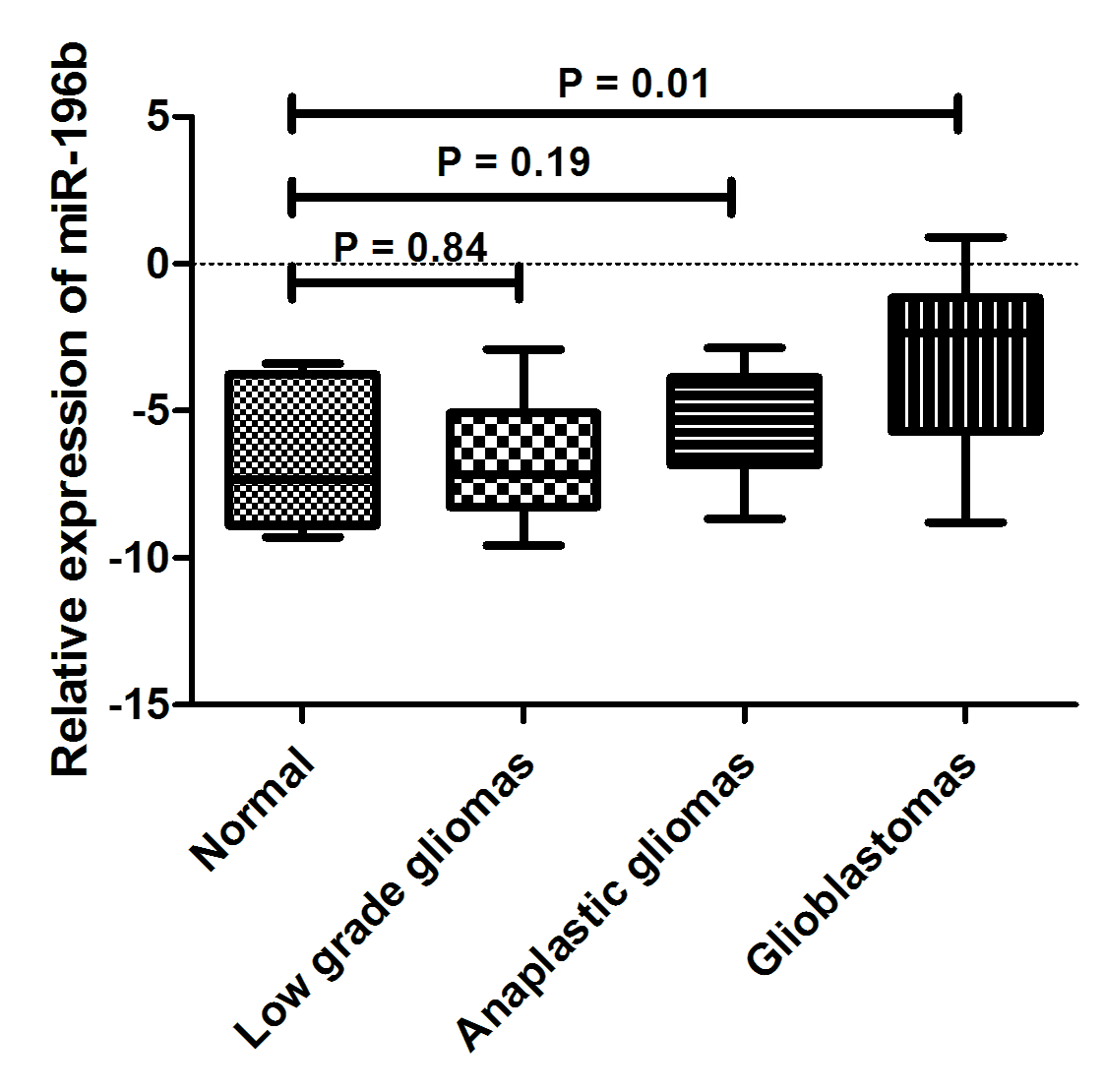

Supplement: Figure S1 — MiR-196b expression levels in 10 Normal tissues, 15 low grade gliomas, 15 anaplastic gliomas and 15 glioblastomas were evaluated by real-time qRT-PCR. And we found that the differential expression of miR-196b between normal tissues and low grade and anaplastic gliomas is not statistical significant (P = 0.84 for low grade gliomas; P = 0.19 for anaplastic gliomas). However, normal tissues have a significant lower of miR-196b when compared Glioblastoma tissues (P = 0.01). (TIF) [file pone.0038096.s001.tif]
